# Seasonal Influenza A H1N1pdm09 Virus and Severe Outcomes: A Reason for Broader Vaccination in Non-Elderly, At-Risk People

**DOI:** 10.1371/journal.pone.0165711

**Published:** 2016-11-10

**Authors:** Elisa Minchole, Ana L. Figueredo, Manuel Omeñaca, Carolina Panadero, Laura Royo, Jose J. Vengoechea, Sergio Fandos, Francisco de Pablo, Salvador Bello

**Affiliations:** 1 Servicio de Neumología, Hospital Universitario Miguel Servet, Zaragoza, Spain; 2 Servicio de Microbiología, Hospital Universitario Miguel Servet, Zaragoza, Spain; Ministry of Health of Peru, PERU

## Abstract

**Background:**

Recent pandemics of influenza A H1N1pdm09 virus have caused severe illness, especially in young people. Very few studies on influenza A H1N1pdm09 in post-pandemic periods exist, and there is no information on the severity of both seasonal influenza A(H1N1) and A(H3N2) from the same season, adjusting for potential confounders, including vaccine.

**Methods and Results:**

We performed a retrospective observational study of adults hospitalized during the 2014 season with influenza A(H1N1) or A(H3N2). All patients underwent the same diagnostic and therapeutic protocol in a single hospital, including early Oseltamivir therapy. We included 234 patients: 146 (62.4%) influenza A(H1N1) and 88 (37.6%) A(H3N2). A(H1N1) patients were younger (p<0.01), developed more pneumonia (p<0.01), respiratory complications (p = 0.015), ARDS (p = 0.047), and septic shock (p = 0.049), were more frequently admitted to the ICU (p = 0.022), required IMV (p = 0.049), and were less frequently vaccinated (p = 0.008). After adjusting for age, comorbidities, time from onset of illness, and vaccine status, influenza A(H1N1) (OR, 2.525), coinfection (OR, 2.821), and no vaccination (OR, 3.086) were independent risk factors for severe disease.

**Conclusions:**

Hospitalized patients with influenza A(H1N1) were more than twice as likely to have severe influenza. They were younger and most had not received the vaccine. Our findings suggest that seasonal influenza A(H1N1) maintains some features of pandemic viruses, and recommend wider use of vaccination in younger adult high-risk patients.

## Introduction

The 2009 influenza pandemic due to the influenza A H1N1pdm09 virus has been considered relatively mild when compared with previous influenza pandemics [1], and even when compared with seasonal epidemics in the community [2]. Some studies have estimated pandemic mortality to have been similar to that of seasonal influenza [3,4], but it has been suggested that this is understated, mainly due to major regional differences in fatalities [5]. Between 62% and 85% of pandemic respiratory deaths were attributed to persons under 65 years (19% in the pre-pandemic period), and more than three-quarters of the deaths associated with influenza A H1N1pdm09 occurred in patients aged between 18 and 64 years [6]. Therefore, many more life-years were lost [5,7]. Clear geographic variations in mortality were evident, with approximately 20-fold more fatalities occurring in Central and South America than in Australia, New Zealand and Europe [7].

Hospitalizations [8,9] and severe cases[1,10] were also higher than expected in young people, and risk of complications such as pneumonia, respiratory failure, shock, sepsis, need for ICU or death in those under 40 years of age were between 2.5 and 4 times higher than in seasonal influenza [1].

During a pandemic, due to a limited preexisting immunity, an increased risk of severe respiratory complications may be expected [1]. However, cross-reactive immunity among adults over 60 years may have contributed to relatively low levels of infection with influenza A H1N1pdm09 in older adults [11]. By between 2010 and 2012, approximately 50% of the population had already developed immunity against the virus [12], and more severe cases in older and people with comorbidities were observed in the following [13]. In the 2009 pandemics, only 48% of hospitalized patients suffered from comorbidities, whereas in the 2010–2012 period, this figure had risen to 75% [14]. However, the range of comorbidities associated with severe disease has not changed significantly in recent years [15]. All these changes, and the replacement of previous seasonal influenza A(H1N1), reflect the evolution of influenza A H1N1pdm09 from a pandemic to a seasonal virus [14].

Some studies searching for risk factors for severe outcome in seasonal influenza have been published. However, it has recently been pointed out that evidence is scarce and adjustment for cofounders lacking [16], especially for vaccine and delayed administration of antiviral drugs after the onset of symptoms [17,18].

A higher proportion of hospitalized patients with severe disease and fatal cases with influenza A(H1N1) have been found in the first post-pandemic seasons, 2010–2011 [3,19] and 2012–13 [13]. However, it remains unclear whether influenza A(H1N1) is more virulent than other seasonal viruses, as reported in specific populations in pandemics, such as young people, the obese, and pregnant women [20–22]. Despite its more aggressive course in these adult populations, some studies performed during and after the 2009 pandemic reported no differences in clinical outcomes attributable to influenza A H1N1pdm09 virulence, compared with seasonal influenza A(H3N2 and non-pH1N1) and B [3,23–25], whereas other studies did find such differences [1,26–28]. Most of them used population-based surveillance data and compared influenza A H1N1pdm09 with seasonal influenza virus from previous influenza seasons, including important potential biases. Only one [28] compared influenza A(H1N1) with A(H3N2) and B viruses in the same season (2010–2011). It contained a high number of adults, but the model did not include important potential confounders such as immunosuppressive conditions and, particularly, vaccination status.

Because of social alarm following admission to the ICU of 6 some otherwise healthy patients in the first week of the influenza season in our hospital, implementation of systematic Influenza searching and management from the emergency department (ED) in 2014 allowed us to study both influenza A viruses in the same season, in a homogeneously immunized population, and with the same diagnostic and therapeutic behavior, including antiviral treatment from the moment influenza was suspected. Our goal was to determine whether seasonal influenza A(H1N1) was associated with a more severe outcome than A(H3N2), after adjusting for every potential confounding factor, including influenza vaccine status, and to assess the impact of vaccination in seasonal A(H1N1) severe influenza.

## Material and Methods

### Study Setting

From 1 January to 30 April 2014, all adult patients (≥ 18 years) admitted to Hospital Universitario Miguel Servet, Zaragoza (Spain) with suspicion of influenza underwent PCR testing of an oropharyngeal swab for influenza A(H1N1), A(H3N2) or B infection. A retrospective observational study of those with positive test results was performed. At that time, a hospital emergency protocol was applied, and all those patients were subject to the same written diagnostic and therapeutic management.

### Definitions

A case of influenza-associated hospitalization was defined as an adult (>18 years) who was admitted with suspected influenza and positive PCR test. Suspicion of influenza included at least one of the IDSA conditions [29]: a) fever and acute onset of respiratory symptoms; b) fever and exacerbation of underlying chronic lung disease; c) elderly with new or worsening respiratory symptoms, including exacerbation of congestive heart failure or altered mental status, with or without fever; d) severely ill persons with fever or hyponatremia. Hospitalized adults admitted without fever and acute respiratory symptoms that developed febrile respiratory illness after hospital admission were not included.

The protocol included a pharyngeal swab at the ED within 6 hours of arrival, and initiation of Oseltamivir therapy as soon as influenza was suspected. Laboratory confirmation was defined as a positive reverse transcription polymerase chain reaction (PCR) result. We used a rapid commercial real-time PCR test (GeneXpert Xpert Flu assay, Cepheid, Sunnyvale, CA, USA).

The protocol included information on demographic characteristics, comorbidities, medical history, influenza vaccination status, clinical course, laboratory analysis, chest X-ray, treatment and outcomes. If any information was not available in the retrospective study, it was collected by reviewing patients’ medical charts and medical records, and by contacting patients or their family members by telephone after discharge. CRP and PCT levels were obtained in the first blood sample.

Obesity was defined as a body mass index of over 30 Kg/m^2^. Immunosuppression included immunoglobulin deficiency, current therapy associated with neoplasia, transplant, human immunodeficiency virus infection/AIDS, or chronic treatment with oral corticosteroids (≥20 mg prednisone/day). The date of influenza vaccination was obtained from the medical record or by phone.

Definition of CAP was based on current Infectious Disease Society of America (IDSA)/ American Thoracic Society (ATS) guidelines, and pneumonia severity was assessed using the PSI and CURB65 scores.

Severe disease was defined as the presence at least of one of the following variables: multilobar or bilateral pneumonia, septic shock, ICU stay, need for invasive mechanical ventilation (IMV), acute respiratory distress syndrome (ARDS) and intra-hospital death. Respiratory complication was defined as the presence of one or more of the following situations: respiratory failure, pneumonia, ARDS, and others (pulmonary embolism, pneumothorax, COPD exacerbation, asthma exacerbation, and acute respiratory failure).

Coinfection was considered when any potential pathogenic bacteria was isolated in blood culture, high-quality respiratory specimen (sputum, bronchial aspirate, BAL) or urinary antigen during hospitalization.

Those with an *a priori* increased risk of severe disease, including age over 65 years, underlying medical conditions, obesity and pregnancy, were considered as high-risk patients.

All influenza vaccines sold in Spain in the 2013–14 season were trivalent, and contained one B virus and two A viruses (A/California/7/2009 (H1N1) pdm09, and A/Victoria/361/2011 (H3N2).

The study was approved by the Ethics Committee -Comité Ético de Investigación Clínica de Aragón- (CEICA). No written informed consent was obtained (retrospective study). Patients records and information was anonymized and de-identified prior to analysis.

### Statistical Analysis

Descriptive analyses were used to summarize patients’ demographic and clinical characteristics, such as age, sex, presence of underlying chronic medical conditions, influenza virus type and subtype, length of hospital stay, intensive care unit (ICU) admission, assisted ventilation and others. Categorical variables were compared using the χ^2^ test or Fisher´s exact test where necessary. Quantitative variables were analyzed using the *t* test or Mann-Whitney *U* test where necessary. Previously, distribution of the data was analyzed using the Kolmogorov-Smirnov test. A multivariable regression analysis was performed to determine whether virus type/subtype was an independent predictor of influenza severity and to explore other potential clinical risk factors. Variables known to be potential confounders (age, comorbidities, influenza vaccination and days from onset of symptoms), as well as those that were associated with disease severity in the univariable analysis (p<0,05) [virus type (H1N1 vs H3N2), coinfection and pregnancy] were included in the multivariable regression model. A p value of less than 0.05 was considered statistically significant. The model diagnostics for the regression analysis were checked using the Hosmer and Lemeshow test.

## Results

A total of 410 adults with possible influenza who visited the ED underwent the PCR test that was performed within 6 hours of arrival and results were obtained in the following 24 hours. Of these, 234 were hospitalized with positive results, and included 146 (62,4%) who were infected with influenza A(H1N1), and 88 (37,6%) with influenza A(H3N2). One other patient was infected with both A viruses and was excluded. No patients were diagnosed with influenza B virus infection. A few cases of influenza A virus infection, who were hospitalized before 1 January and some cases considered as nosocomial influenza were not included. The highest incidence of hospitalization occurred in January, and February was the only month with more cases of A(H3N2).

Oseltamivir was initiated before PCR results were obtained, within 24 hours after admission, and then withdrawn in negative cases (except those who had been in close contact with true influenza patients). In positive cases, therapy was continued for at least 7 days at the usual doses.

**Fig 1:**
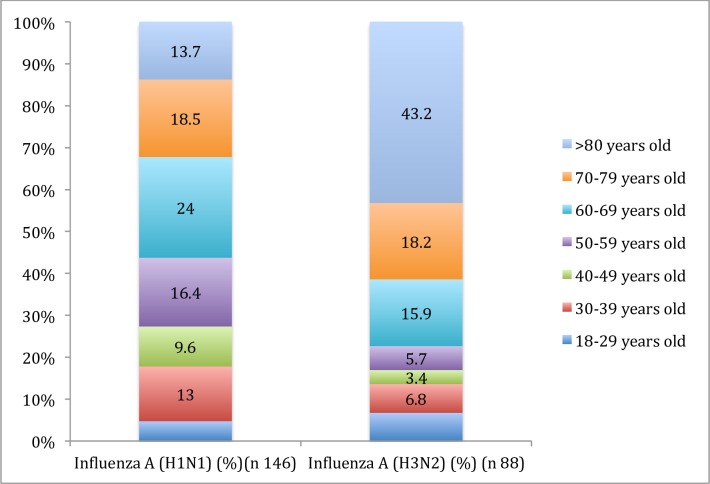
Viral infection A(H1N1) vs A(H3N2) by age group.

The median age was 64 years in influenza A(H1N1), and 77 years in A(H3N2) cases (P<0.001). The largest proportional A(H1N1) age group for influenza A(H1N1) was 60–65 years (24%), whereas for A(H3N2), it was >80 years (43.2%). Age distribution was very different for both viruses. Fig 1.

Most of patients (76.5%) had at least one underlying medical condition, with no differences between both groups. Influenza A(H1N1) patients had a higher rate of immunosuppression, while those with A(H3N2) had more frequent underlying cerebrovascular disease, and chronic heart disease. Table 1.

**Table 1:** Underlying medical conditions in patients with influenza A(H1N1) and A(H3N2) virus.

Comorbidities	Influenza A(H1N1) (n = 146)	Influenza A(H3N2) (n = 88)	P
Body mass index (>30)	26 (17.8%)	11 (12.5%)	0.290
Diabetes	29 (19.8%)	20 (22.7%)	0.602
Chronic liver disease	8 (5.5%)	3 (3.4%)	0.469
Chronic renal failure	22 (15.1%)	13 (14.8%)	0.951
**Chronic heart disease**	**34 (23.3%)**	**31 (35.2%)**	**0.048**
**Cerebrovascular disease**	**12 (8.2%)**	**19 (21.6%)**	**0.003**
COPD	31 (21.2%)	12 (13.6%)	0.146
Asthma	12 (8.2%)	9 (10.2%)	0.603
Neoplasia	26 (17.8%)	8 (9.1%)	0.067
Bronchiectasis	4 (2.7%)	2 (2.3%)	0.827
Pregnancy	17 (11.6%)	5 (5.7%)	0.130
**Immunosuppression (1)**	**37 (25.3%)**	**9 (10.2%)**	**0.005**
≥ 3 Comorbidities	38 (26.0%)	20 (22.7%)	0.496
No comorbidities	36 (24.6%)	19 (21.6%)	0.592

(1) Immunosuppression: HIV, active neoplasia, active chemotherapy, transplant, chronic treatment with oral corticosteroids.

The median time from onset of illness to hospital admission was 4.4 days in the influenza A(H1N1) group and 3.3 days in the A(H3N2) group (p = 0.024). The most common symptoms were cough and fever, with no significant differences in clinical signs and symptoms between the groups. Table 2.

**Table 2:** Clinical manifestations, radiological and laboratory findings and outcomes of patients with influenza A(H1N1) and A(H3N2) virus infection.

	Influenza A(H1N1) (n = 146)	Influenza A(H3N2) (n = 88)	P
Clinical manifestations, no (%)			
**Days since onset of symptoms (SD)**	**4.49 (±4.244)**	**3.46 (±2.571)**	**0.024**
**Vaccination (n = 191) (%)**	**48/118 (40.7%)**	**44/73 (60.3%)**	**0.008**
**Age (median)**	**64**	**77**	**<0.001**
Sex (women/men)	69/77	48/40	0.280
Fever at admission	119 (81.5%)	75 (85.2%)	0.132
Cough	125 (85.6%)	73 (82.9%)	0.781
Expectoration	94 (64.4%)	49 (55.7%)	0.235
Dyspnea	98 (67.1%)	61 (69.3%)	0.783
Pleuritic chest pain	27 (18.5%)	10 (11.4%)	0.161
Gastrointestinal disorders	23 (15.8%)	18 (20.4%)	0.342
Headache	18 (12.3%)	9 (10.2%)	0.656
Myalgias	36 (24.6%)	18 (20.5%)	0.499
Sore throat	17 (11.6%)	8 (9.1%)	0.567
Radiological findings			
**Pneumonia**	**66 (45.2%)**	**14 (15.9%)**	**<0.001**
- Alveolar opacities	43/66 (65.1%)	12/14 (85.7%%)	0.132
- Multilobar infiltrates	38/66 (57.6%)	6/14 (42.8%)	0.399
- Bilateral infiltrates	30/66 (45.4%)	5/14 (33.3%)	0.470
- PSI score IV-V	34/66 (51.5%)	9/14 (64.3%)	0.384
- CURB65 score >2	31/66 (46.9%)	7/14 (50%)	0.837
Clinical outcomes, no (%)			
**- Respiratory complications**	**98 (67.1%)**	**45(51.1%)**	**0.015**
- Renal failure	21 (14.4%)	7 (7.9%)	0.142
- Heart failure	35 (23.9%)	22 (25%)	0.859
- Neurologic complications	8 (5.5%)	1 (1.1%)	0.098
- Digestive complications	8 (5.5%)	10 (11.3%)	0.099
- ARDS (*)	**7 (4.8%)**	**0 (0%)**	**0.047**
**- Septic shock**	**16 (10.9%)**	**3 (3.4%)**	**0.049**
**Intensive care unit admission**	**23 (15.7%)**	**5 (5.7%)**	**0.022**
NIMV (**)	12 (8.2%)	7 (7.9%)	0.943
IMV (***)	**18 (12.3%)**	**4 (4.5%)**	**0.049**
Hospital stay, median days(range)	8 (1–84)	9 (1–84)	0.856
Death during hospitalization	16 (19.9%)	9 (10.2%)	0.870
Death at 30 days	19 (13%)	11 (12.5%)	0.920
Death at 180 days	26 (17.8%)	14 (15.9%)	0.489
Death at 365 days	29 (19.8%)	15 (17%)	0.702
Bacterial coinfection no, (%)	24 (16.4%)	9 (10.2%)	0.194
Laboratory findings			
**Leukopenia (<4,000/mm**^**3**^**), no (%)**	**18 (12.3%)**	**4 (4.5%)**	**0.044**
**Leukocytosis (>12,000/mm**^**3**^**), no (%)**	**42 (28.7%)**	**10 (11.4%)**	**0.020**
Neutropenia (<500/mm3), no (%)	3 (2.1%)	1 (1.1%)	0.589
**Anemia (Hematocrit <36%), no (%)**	**40 (27.4%)**	**13 (14.7%)**	**0.030**
**Thrombocytopenia (<150.10**^**3**^**/mm**^**3**^**), no (%)**	**43 (29.5%)**	**39 (44.3%)**	**0.026**
Hyponatremia<135mmol/l, no (%)	49 (33.5%)	22 (25%)	0.120
Serum creatinine >1.5 mg/dl, no (%)	24 (16.4%)	10 (11.4%)	0.292
C-reactive protein (CRP) (mg/dl) (range) (N = 94)	11.82 (0–46) (n = 60)	9.38(1–46) (n = 34)	0.256
Procalcitonin (ng/ml) (PCT) (range) N = 85	0.14 (0–84) (n = 62)	0.13 (0–35) (n = 23)	0.793

(*) ARDS: Adult respiratory distress syndrome

(**) NIMV: Non-Invasive mechanical ventilation

(***) IMV: Invasive mechanical ventilation

Pneumonia, respiratory complications, ICU admission, ARDS, septic shock, and need for IVM were more common in the influenza A(H1N1) group. Pneumonia severity was assessed at admission (PSI and CURB65 scores), and no statistical differences were found between the two groups. Only one patient developed an encephalopathy secondary to the flu and recovered. Intrahospital mortality was higher for influenza A(H1N1), but not statistically different. Biomarker levels showed no statistical differences between the two groups. The main clinical manifestations, radiological and analytical findings and outcomes are shown in Table 2. Causes of intrahospital mortality are summarized in Table 3.

**Table 3:** Causes of mortality in both influenza A(H1N1) and A(H3N2) groups.

Causes of mortality	A(H1N1) (n = 16)	A(H3N2) (n = 9)
ARDS (*)	4	
Pneumonia. Multiorgan failure. Septic shock.	3	4
Septic shock. Heart disease.	3	
Heart disease	2	2
Heart disease. Renal disease.		1
Renal disease. Rectal bleeding.	1	1
Neutropenia. Multiorgan failure.	1	
Airway aspiration		1
Bilateral pneumonia. Pneumothorax.	1	
Lung disease. Bowel perforation.	1	

(*) ARDS: Adult respiratory distress syndrome

The bacterial co-infection rate was similar in both groups. The only sterile fluid analyzed was blood because the physician in charge did not consider sampling either pleural (8 cases) or cerebrospinal fluid to be necessary. *Streptococcus pneumoniae* and *Pseudomonas aeruginosa* were the pathogens most frequently found. Both PCT and CRP levels were higher in the case of bacterial involvement than in isolated viral infection (Table 4), and severe cases also showed increased levels.

**Table 4:** C-Reactive Protein and Procalcitonin levels at admittance in bacterial coinfection vs isolated virus infection.

	Coinfection (viral+bacterial)	Viral	P
C-reactive protein (mg/dl) (range) (N = 93)	24.9 (1–46)	9.91(0–42)	0.015
Procalcitonin (ng/ml) (range) N = 84	0.99 (0–14)	0.11 (0–84)	0.001

Sixty-eight out of 234 patients (29%) were considered severe cases. Of these patients, 52 (76.5%) were associated with influenza A(H1N1), and 16 (23.5%) with A(H3N2) (p = 0.005). Disease severity was also associated with bacterial coinfection (p<0.001). There was no statistically significant association between age or vaccination and severe influenza in the univariate analysis. Among individually selected underlying medical conditions, influenza severity was significantly lower among pregnant women (p <0.028). Table 5.

**Table 5:** Characteristics associated with influenza disease severity for hospitalized adults.

Characteristic	Non severe (n = 166) No. (%)	Severe (n = 68) No (%)	P value
**Virus type/subtype**			**0.005**
Influenza A(H1N1)	94 (56.6%)	52 (76.5%)	
Influenza A(H3N2)	72 (43.4%)	16 (23.5%)	
Days since onset of symptoms (SD)	4.05	4.22	0.764
Vaccination status (n = 191)	73/142 (51.4%)	19/49 (38.7%)	0.129
Vaccination status in high-risk patients (n = 174)	71/130 (54.6%)	19/44 (43.2%)	0.190
Age median (±SD)	62.5 (±20.6)	65.6 (±14.6)	0.263
Sex			0.150
Male	78 (46.9%)	39 (57.4%)	
Female	88 (53.1%)	29 (42.6%)	
Body mass index ≥30	26 (15.6%)	11 (16.2%)	0.674
**Pregnant (n = 22)**	**21 (12.6%)**	**1 (1.5%)**	**0.028**
Underlying medical conditions			
Comorbidities ≥3	20 (12%)	7 (10.3%)	0.703
Lung disease (**)	43 (25.9%)	24 (35.3%)	0.149
COPD (***)	28 (16.8%)	15 (22.1%)	0.353
Asthma	15 (9%)	6 (8.8%)	0.959
Bronchiectasis	3 (1.8%)	3 (4.4%)	0.360
Heart disease	50 (30.1%)	15 (22.1%)	0.213
Renal disease	23 (13.8%)	12 (17.6%)	0.461
Liver disease	7 (4.2%)	4 (5.8%)	0.586
Cerebrovascular disease	24 (14.5%)	7 (10.3%)	0.396
Neoplasia	24 (14.5%)	10 (14.7%)	0.961
Immunosuppression	33 (19.8%)	13 (19.1%)	0.894
**Coinfection**	**14 (8.5%)**	**19 (27.1%)**	**<0.001**
**C-Reactive protein (mg/dl) median (±SD)**	**7.45 (±6.8)**	**18.63 (±13.2)**	**<0.001**
**Procalcitonin (ng/ml) median (±SD)**	**0.42 (±1.57)**	**9.06 (±19.5)**	**0.003**

(***) Lung disease: COPD, bronchiectasis and asthma.

(**) COPD: chronic obstructive pulmonary disease.

A multivariate analysis was performed to search for independent predictors of severity. Age, etiology, vaccination status, comorbidity (COPD, asthma, bronchiectasis, heart, cardiovascular, renal and liver chronic diseases, active neoplasia and immunosuppression), days from onset of illness to admission, and bacterial coinfection were included in the model. Biomarkers were not included in the final model because of the small number of patients for whom these data were available. Independent predictors for severity included no vaccine (OR, 3.086; p = 0.013), influenza A(H1N1) etiology (OR, 2.525; p = 0.037), and bacterial coinfection (OR, 2.821; p = 0.044). Table 6. The model diagnostics for regression analysis were checked using the Hosmer and Lemeshow test (p = 0.751). Our model with three significant variables (etiology, vaccination status and coinfection) was able to predict the severity in 73.4% of patients, with no difficulties in the iterative estimation algorithm. The sensibility was 14.3% and the specificity was 95.7%.

**Table 6:** Logistic Regression analysis of factors associated with severe influenza among adults in influenza season.

	NONADJUSTED		ADJUSTED		
Characteristic	OR	p-value	OR	95%CI	p-value
Age	1.009	0.263	1.019	0.989–1.050	0.216
**Etiology [(Influenza A(H1N1) vs A(H3N2)]**	**2.487**	**0.005**	**2.525**	**1.058–6.024**	**0.037**
**Vaccination status**	**0.599**	**0.129**	**0.324**	**0.132–0.792**	**0.013**
**Coinfection (viral +bacteria)**	**4.099**	**<0.001**	**2.821**	**1.030–7.723**	**0.044**
Days since onset of symptoms	1.012	0.764	1.046	0.856–1.068	0.425
Heart disease	0.657	0.213	0.499	0.193–1.287	0.150
COPD	1.395	0.353	1.281	0.486–3.377	0.616
Asthma	0.974	0.959	1.486	0.383–5.762	0.567
Cerebrovascular disease	0.679	0.396	1.164	0.379–3.573	0.791
Renal failure	1.332	0.461	1.965	0.662–5.830	0.224
Liver disease	1.420	0.586	0.637	0.101–4.032	0.632
Neoplasia	1.020	0.961	0.986	0.333–2.926	0.980
Immunosuppression	0.953	0.894	0.635	0.219–1.840	0.403
Pregnancy	0.103	0.028	0.152	0.015–1.503	0.107
Obesity	1.190	0.674	0.903	0.331–2.462	0.843
Comorbidities (≥3)	0.838	0.703	1.023	0.213–4.921	0.978

Forty-eight per cent (92/191) of patients had received influenza vaccination. Vaccination was more frequent in the influenza A(H3N2) group than in the A(H1N1) group (60.3% vs 40.7%; p = 0.008). Table 2. According to the recommendations of the Spanish Ministry of Health, flu vaccine was indicated in 91% (174/191) of our influenza subjects, and about 40% of subjects with high-risk conditions were not covered by the vaccine. Table 7. The most common reason for vaccination was being over 65 years old. Only 1/16 (6.25%) pregnant woman and 13/34 (38.3%) immunosuppressed patients were vaccinated. Table 7. In the influenza A(H1N1) group, severe disease was found in 20.8% of vaccinated patients and in 38.6% of nonvaccinated patients (p = 0.041). In the influenza A(H3N2) group, there were no statistical differences in vaccination and poor clinical outcome. Table 5.

**Table 7:** Indications and influenza vaccination status before hospital admission.

Indication of vaccination (n)	Vaccination n (%)
≥65 years old (114)	79/114 (69.3%)
Pregnancy (16)	1/16 (6.25%)
COPD (38)	26/38 (68.4%)
Asthma (16)	12/16 (75%)
Heart disease (58)	37/58 (63.7%)
Immunosuppression (34)	13/34 (38.3%)
Renal disease (30)	20/30 (66.6%)
BMI ≥ 30 (29)*	17/29 (58.6%)
Diabetes mellitus (44)	28/44 (63.6%)
TOTAL (379)	233/379 (61.4%)

(*) BMI: body mass index.

## Discussion

Our study suggests that during the fourth influenza season after the 2009 pandemic (2013–2014), hospitalized adults with influenza A(H1N1) were more than twice as likely to have severe influenza (death, ICU, IVM, ARDS, septic shock, multilobar pneumonia) as patients hospitalized with A(H3N2) infection. This was true even after controlling for age, comorbidity, days after onset of symptoms, bacterial coinfection and vaccination status (OR, 2.525). We did not adjust for antiviral drugs because all patients were systematically treated with Oseltamivir at the moment of clinical suspicion, even before confirmation of diagnosis, and always within 24 hours of arrival at the ED. Our model diagnostics with three covariates (etiology, coinfection, and vaccination status) were able to predict the severity in 73.4% of patients. This model showed a high specificity and low sensibility. It may therefore be used to identify patients with lower severity and help clinicians to make decisions regarding management of these patients. More studies with other populations are necessary to verify the validity of the model.

During pandemics, influenza A H1N1pdm was found to be associated with particular severity in some populations, such as young people, the obese, and pregnant women [17,20–22]. However, information regarding its possible greater virulence when comparing clinical outcomes with that of seasonal influenza A(H3N2) virus, in studies done during and after the 2009 pandemic [1,3,23–28,30] is the subject of debate. Most of those studies did not control for factors that may impact influenza disease severity, such as age and underlying medical conditions. They were performed using population-based surveillance data and compared influenza A(H1N1) with seasonal influenza viruses from previous influenza seasons. This may include potential biases, such as varying medical care-seeking behavior, host immune adaptation, degree of matching between circulating and vaccine-selected viruses, and likelihood of being prescribed antiviral drugs. To our knowledge, ours is the second study to examine seasonal severity of both influenza A viruses in the same season in hospitalized patients. The first one [28] compared influenza A(H1N1) with A(H3N2) and B viruses (2010–2011), and included 2498 adults from 276 hospitals. Data were collected from the Influenza Hospitalization Surveillance Network (FLUSORV-NET) to perform a multivariate analysis adjusted for age, underlying medical conditions, and antiviral therapy, to identify predictors for severity (ICU admission or death) associated with these viruses. They found that influenza A(H1N1) patients were twice as likely to have severe influenza as patients with A(H3N2) or influenza B. However, their model did not include major potential confounders such as immunosuppressive conditions and, especially, vaccination status.

Our study suggests greater virulence of influenza A(H1N1) compared with A(H3N2) in hospitalized subjects, 4 years after pandemics. A more important extension to the lower respiratory tract resulting in severe and even fatal pneumonia has been associated with influenza A(H1N1) compared to seasonal viruses [31, 32].

Other independent factors associated with severe outcome were coinfection and lack of previous vaccine administration. Bacterial coinfection is recognized as a factor that leads to increased morbidity and mortality from influenza virus infections [33–36]. *Streptococcus pneumoniae* and *Pseudomonas aeruginosa* were the most frequently involved bacteria in coinfections in our series, mostly in patients with worst outcomes and related with influenza A(H1N1) infection. These bacteria were most commonly isolated as coinfecting pathogens in some recent influenza A(H1N1) cohorts of hospitalized patients with pneumonia [35,36]. It is true that, in this case, it is difficult to consider bacterial coinfection either as predictor or as outcome, because many bacteria were isolated some days after admission. Influenza viruses have recently been found to promote susceptibility to lethal bacterial coinfection, independently of pathogen burden or inflammatory response [34].

It has been established that vaccination may provide protection from severe illness requiring hospitalization [37,38]. Advanced age, chronic illness, as well as obesity, third trimester of pregnancy and postpartum period, are considered to increase the risk of severe outcomes, and these groups should be the main targets for influenza vaccination. Due to the limited supply of vaccines, the WHO and most countries prioritize specific risk groups. Although some recommendations are consistent, such as vaccination of healthcare workers, pregnant women, and those with certain high-risk conditions, there are also discrepancies, such as the age groups that need to be prioritized. In Spain, people over 65 years of age very often receive the vaccine, although there is also an indication for people 65 years of age with chronic diseases and conditions, including pregnancy and morbid obesity [17]. In our group, nearly 70% of those aged over 65 years were vaccinated, and the rate of vaccination among those with influenza A(H3N2) infection (median age, 77), was 60.3%. However, in influenza A(H1N1) patients (median age, 64), this figure was only 40.7% (p = 0.008). The rate of comorbidities was similar in both groups (78.4% vs 75.5%); most of our cases from both groups were therefore people with underlying conditions. The low vaccination rate among some of our high-risk patients, such as pregnant and immunosuppressed patients, was remarkable. Significant differences were observed in the rate of severe cases from influenza A(H1N1) between vaccinated and non-vaccinated patients. Therefore, patients infected with influenza A(H1N1) were younger than those infected with A(H3N2), had received less vaccination despite a similar rate of comorbidities, and showed more severe outcomes associated with influenza A(H1N1). Two recent studies have shown a relationship between low rate of vaccination and critical illness [39] and the need for ICU admission in young people [40] infected with influenza A(H1N1). An early low anti-A(H1N1) antibody response in hemagglutination-inhibition was associated with fatal cases [40]. Associated comorbidities were different: immunosuppression was more common in influenza A(H1N1), and cardiovascular disease in A(H3N2). All these findings suggest the need for a higher rate of vaccination in young adults, especially those with underlying medical conditions. A recent study of hospitalized influenza pneumonia patients during two seasons (2010–2012) found an estimated vaccine effectiveness of 56.7%, and higher for influenza A(H1N1) (59.5%) than A(H3N2) (45.1) [41]. However, according to Eurosurveillance data, the influenza vaccine effectiveness (VE) for the season 2013/2014 in Spain was of 35% [33% for A (H1N1) and 28% for A (H3N2)], a lower percentage in comparison with previous years. It is true that, in previous seasons, influenza VE was consistently lower in Spain than in the US and Canada, possibly due to different characteristics of the viruses, according to the Spanish Influenza Sentinel Surveillance System (SISS) (antigenic tests are not yet available in our country) [42].

Despite the younger age of the subjects, influenza A(H1N1) was associated with more severe outcomes. Similar findings have been reported both during pandemics [26,30] and in post-pandemic seasons [28], in studies in which both viruses were not compared in the same year. Both a decremental pattern of severe diseases in those aged under 65 years lasting up to a decade after the pandemic [43] and a similar epidemiology to that of seasonal influenza A(H3N2) and A (H1N1) virus have been anticipated for influenza A H1N1pdm [44]. Our study and others on the 2010–2011 post-pandemic period [3, 19] and in the 2012–2013 seasons [13] found a higher proportion of hospitalized severe cases due to influenza A(H1N1) that were older than in pandemics, but younger than in seasonal influenza, and frequently with underlying medical conditions [3, 13]. For the moment, the age distribution and clinical spectrum of seasonal influenza A(H1N1) remains different from those of A(H3N3) 4 years after the 2009 pandemics. Knowledge of circulating viruses and their epidemiologic parameters helps to understand the burden of seasonal and pandemic influenza, and allows quantification and comparison of the risk of severe outcomes [45]. These findings reinforce the need to identify and protect groups at highest risk [3].

Patients with influenza A(H1N1) infection came to hospital longer after onset of symptoms than those with A(H3N2) (4.49 days vs 3.46 days), probably due in part to age differences. This circumstance may have had some impact on outcomes. However, initiation of Oseltamivir therapy, even after the second day from onset of illness, has been found to decrease mortality [18]. Furthermore, this difference was not found to be an independent risk factor for severity in our multivariate model, and in any case, it was not possible to administer antiviral drugs earlier.

The most important limitation of our study is the relatively small number of cases, from a single hospital. However, this fact allowed us to avoid several important potential biases from previous studies from medical records, including potential changes in coding practices, influenza tests performed at the discretion of the treating clinicians [1], different influenza diagnostic tests, and heterogeneous diagnostic and therapeutic behavior between hospitals [28]. Although our data were obtained retrospectively, a similar approach was used for every patient. The apparent “protective” role of pregnancy for severe disease can be explained by the systematic hospitalization of pregnant women, even mild cases, during the first weeks of the season due to social alarm. We did not discriminate between obesity and morbid obesity. A potential effect of corticosteroids in severe influenza infection has not been established [18], and we did not adjust for treatment with corticosteroids and antibiotics. However, all patients were managed using a similar approach. Strengths include the study of both influenza A viruses in the same season, in a homogeneously immunized population, and with the same clinical behavior, including antiviral treatment from the moment influenza was suspected.

We conclude that seasonal influenza A(H1N1) is a risk factor for severe influenza outcome in hospitalized patients 4 years after the 2009 pandemics, continues to affect younger people than influenza A(H3N2), who were less frequently vaccinated. No vaccination was the most powerful predictive factor associated with severe outcome. Our results suggest recommending wider use of vaccines in non-elderly patients at risk of severe influenza.

## Supporting Information

S1 Table TABLA DE GRIPE ARTC1. Complete data base.
(XLSX)
